# Axial spatial distribution focusing: improving MALDI-TOF/RTOF mass spectrometric performance for high-energy collision-induced dissociation of biomolecules

**DOI:** 10.1002/rcm.7458

**Published:** 2015-12-29

**Authors:** O Belgacem, E Pittenauer, M E Openshaw, P J Hart, A Bowdler, G Allmaier

**Affiliations:** 1 Shimadzu, Kratos AnalyticalTrafford Wharf Road, Manchester, M17 1GP, UK; 2 Institute of Chemical Technologies and Analytics, Vienna University of TechnologyGetreidemarkt 9/164, 1060, Vienna, Austria

## Abstract

**Rationale:**

For the last two decades, curved field reflectron technology has been used in matrix-assisted laser desorption/ionization time-of-flight (MALDI-TOF) mass spectrometers, assisting in the generation of post-source-decay (PSD) or collision-induced dissociation (CID) without decelerating precursor ions, producing true high-energy CID spectra. The result was the generation of product ion mass spectra with product ions typical of high-energy (10 keV and beyond) collision processes. The disadvantage of this approach was the lack of resolution in CID spectra resulting from the excess laser energy deposition used to generate those MS/MS spectra. The work presented in this study overcomes this limitation and includes comprehensive examples of high-energy and high-resolution CID MALDI-MS/MS spectra of biomolecules.

**Methods:**

The devices used in this study are TOF/RTOF instruments equipped with a high-vacuum MALDI ion source. High-resolution and high-energy CID spectra result from the use of axial spatial distribution focusing (ASDF) in combination with curved field reflectron technology.

**Results:**

A CID spectrum of the P_14_R_1_ peptide exhibits product ion resolution in excess of 10,000 (FWHM) but at the same time yields typical high-energy product ions such as w- and [y–2]-type ion series. High-energy CID spectra of lipids, exemplified by a glycerophospholipid and triglyceride, demonstrate C–C backbone fragmentation elucidating the presence of a hydroxyl group in addition to double-bond positioning. A complex high mannose carbohydrate (Man)_8_(GlcNAc)_2_ was also studied at 20 keV collision energy and revealed further high-energy product ions with very high resolution, allowing unambiguous detection and characterization of cross-ring cleavage-related ions.

**Conclusions:**

This is the first comprehensive study using a MALDI-TOF/RTOF instrument equipped with a curved field reflectron and an ASDF device prior to the reflectron. © 2015 The Authors. *Rapid Communications in Mass Spectrometry* published by John Wiley & Sons Ltd.

The matrix-assisted laser desorption/ionization (MALDI) technique is a soft ionization method known to be particularly well suited for the analysis of large and/or fragile molecules.^1^ Since its discovery in the late 1980s,^2^,^3^ this technique has been coupled to several mass-analyzing systems^4^–^7^ but has been extensively used in combination with time-of-flight (TOF) analyzers due to the pulsed nature of lasers. MALDI mass spectrometry has been used to analyze a wide range of biomolecules and in particular lipids, peptides and oligosaccharides.^8^–^10^ Valuable information, when dealing with the structural elucidation of these biomolecules, can be obtained through sequencing experiments where precursor ions generated in the ion source of the mass spectrometer are isolated using a Bradbury-Nielsen^11^ ion gate for example, and then fragmented. The spectra resulting from the fragmentation processes have been called tandem (MS/MS), post-source decay (PSD) and laser-induced-dissociation (LID) spectra. Experiments in which the precursor ions undergo collisions with a neutral gas, such as helium or argon, give rise to collision-induced dissociation (CID) spectra. Such PSD/LID and CID MS/MS experiments can be performed in most MALDI-TOF/RTOF mass spectrometers, depending on the instrumental parameters and setup. The fragmentation process in mass spectrometry has been studied extensively^12^,^13^ and these investigations are ongoing. Importantly, the fragmentation of biomolecules in most MALDI instruments is induced mainly by the excess of internal energy of the precursor ions obtained during the desorption/ionization event.^14^–^16^ The excess of internal energy is usually obtained from the use of a UV laser during the initial desorption/ionization steps.^17^

Cotter *et al*.^18^ described a mass spectrometer capable of generating high-energy (HE) CID (20 keV lab collision energy) MALDI-MS/MS spectra. This mass spectrometer was equipped with a high-vacuum ion generation chamber, and a collision cell followed by an ion selector prior to a curved field reflectron (CFR). In 2006, Belgacem *et al*.^19^ described a very similar mass spectrometer where the collision cell was placed, this time, after the ion selector (or ion gate). Such a configuration proved to be more efficient for the production of HE-CID ions in product ion spectra. This was believed to be because the metastable ions formed in the source are more likely to undergo HE-CID if the collisions occur later in their metastable decay life-time and thus further away from the ion source. Using the aforementioned MALDI mass spectrometer, it is possible to generate MS/MS spectra with very high-energy collisions when employing helium as a collision gas (these spectra will be referred to as CID spectra). Similarly, MS/MS spectra can be obtained without gas in the collision chamber and, in that case, they can simply be described as PSD mass spectra.^15^ The CID spectra generated in this device^19^ do have some advantages. These include, but are not limited to, the observation of side-chain fragmentation, charge-remote fragmentation, and cross-ring cleavage when studying the three mentioned compound classes.

On its own, the use of CID is not enough to generate significant yields of product ions. Abundant CID spectra can only be obtained if sufficient energy is transferred to the analytes during the desorption/ionization event. This is generally achieved by increasing the laser energy deposited onto the sample.^14^ However, this increase in deposited laser energy results in a significant loss of mass resolution for both the precursor and the product ions. The reduction in mass resolution has led to the development of axial spatial distribution focusing (ASDF)^20^ in order to achieve mass resolution in MS/MS similar to that normally seen for MS and, furthermore, be independent of the increased laser fluence.

The MALDI process results in a plume of material expanding from the sample surface in which the ions are formed during the first tens of nanoseconds. The resultant ions have typical velocities of a few hundred metres per second and trajectories largely normal to the sample surface. Within the plume, there are distributions of velocity and position, which depend on the characteristics of the sample and the laser. These distributions can be resolved into components in two directions, as shown in Fig.1(A): the radial direction parallel to the sample surface and the axial direction normal to the sample surface and also parallel to the ion optical axis. The axial velocity distribution is dependent on the type of matrix and the laser wavelength while the radial velocity distribution is affected by the sample topography and angle of incidence of the laser. The spatial distributions depend on the laser beam diameter in the radial direction and the sample depth as well as laser pulse length in the axial direction.

**Figure 1:**
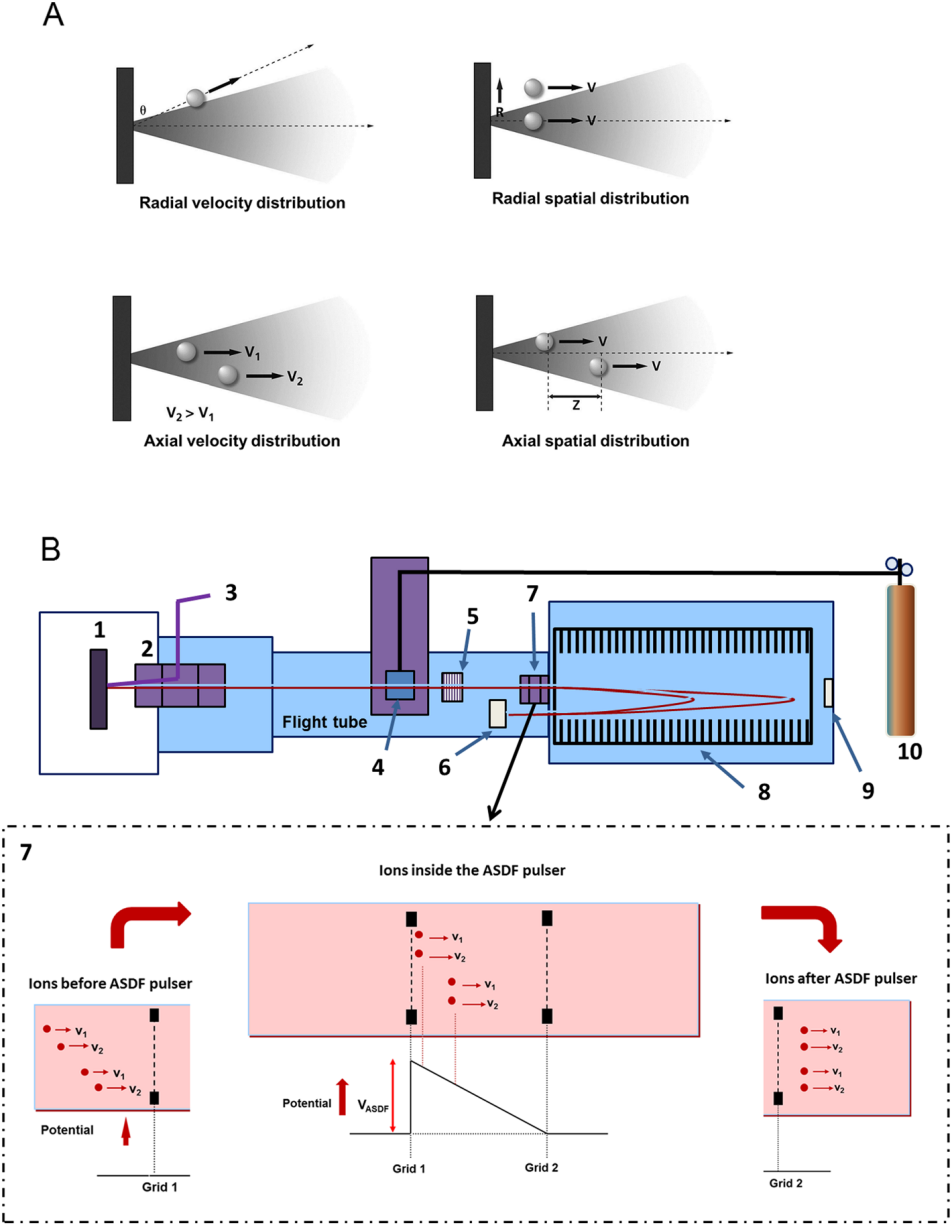
(A) Simplified view representing the various ion distributions in a MALDI source of a mass spectrometer. (B) Schematic and simplified view of the MALDI TOF/ReTOF instrument equipped with an ASDF cell: 1 XY sample stage. 2 Source and ion optics. 3 Nd:YAG Laser. 4 CID cell. 5 Ion gate. 6 Reflectron detector. 7 ASDF cell. 8 Curved field reflectron. 9 Linear detector. 10 Helium gas supply. The inset represents the mechanisms by which ions are re-focused in the ASDF cell.

In terms of their effect on the performance of the mass spectrometer, the radial distributions are responsible for the sensitivity while the axial distributions produce a spread in the time of flight of ions and ultimately determine the mass resolution. Most, but crucially not all, of these distributions are compensated for by the ion source design. All the radial distributions are controlled by electrostatic lenses in the ion optics. The axial velocity distribution is controlled by the pulsed extraction.^21^ However, there is no method of controlling the effect of the axial spatial distribution (that is to say the differences in initial position) of the ions. This is known^22^ to be because pulsed extraction can compensate for either the velocity distribution or the spatial distribution but not both simultaneously.

When the laser fluence is close to the threshold for MALDI ions, the axial spatial distribution can be very small so that its contribution to the time-of-flight spread is also small and high mass resolution is achieved. However, when the laser fluence is increased, as is the case for MS/MS, it is the authors' assertion that the size of the initial axial spatial distribution increases to the point where it dominates the spread in the time of flight and results in low mass resolution.

Since the axial spatial distribution cannot be corrected in the ion source, an additional step is required downstream during the flight of the ions that acts on the axial spatial distribution without disturbing the effect of the pulsed extraction on the axial velocity distribution. This is the function of the ASDF cell and its location is determined by three factors. First, the spatial distribution of the ions along the flight path due to the initial axial spatial distribution has to be very much larger than that due to the initial velocity distribution. Secondly, the ASDF must be carried out in the field-free region where the precursor and product ions are still moving together. Finally, because applying an electrostatic field modifies the ion energies, the ASDF should be after all (or the vast majority) of the product ions have been formed, whether by PSD or CID. The location which satisfies these criteria is just in front of the reflectron, as shown schematically in Fig.1(B).

The ASDF cell, item 7 in the inset of Fig.1(B), consists of two high transmission grids spaced 12.5 mm apart where a fast high-voltage pulse can be applied to the grid 1 closest to the ion source and the exit grid 2 is at ground potential. Initially, grid 1 is at 0 V until the precursor (and product) ions of interest, already selected by the ion gate, enter the ASDF cell. At that time, the high-voltage pulse is applied to the first grid and an axial electrostatic field is produced. The result is pulse bunching of the ions such that the spatial distribution is focused just after the exit of the cell. Because the axial spatial distribution in the ASDF cell is predominantly due to the initial spatial distribution in the ion source, the initial spatial distribution is focused while the axial velocity distribution is largely unaffected. Typically, the ASDF focal length is 50 to 80 mm depending on the *m*/*z* values of the product ions. This range is effectively matched to the transfer characteristics of the curved field reflectron so that the net effect is a narrow time distribution at the detector for both the initial axial velocity and the spatial distributions. The result is high mass resolution for MS/MS and this is largely independent of laser fluence.

In this paper, the effectiveness of ASDF in combination with the CFR will be demonstrated by the results of fragmentation of a variety of biomolecules by PSD and CID.

## Experimental

### Description of the MALDI tandem instruments

Two different instruments were used to record the CID spectra. The first TOF/RTOF device utilized in this work is a MALDI mass spectrometer based on the Axima series instrument equipped with a curved field reflectron (Axima Performance, Shimadzu Kratos Analytical, Manchester, UK). A detailed description of the instrument can be found elsewhere.^19^ Typically, the instrument is equipped with a nitrogen laser (λ = 337 nm) generating a 3 ns pulse width at a maximum pulse rate of 50 Hz. Samples are deposited on a stainless steel microscope slide formated target (FlexiMass, Shimadzu Kratos Analytical) and the MALDI ion source is operated at approximately 10^−6^ mbar. Ions are selected for CID using an ion gate consisting of two Bradbury-Nielsen wire gates.^11^ To perform high-energy CID, a gas is introduced into a differentially pumped collision chamber (helium is used as the collision gas), where the pressure is one order of magnitude higher (typically around low 10^−5^ mbar) and where ions experience 20 keV collisions because no deceleration of the ion beam (20 kV extraction voltage) is performed. High-energy CID spectra at higher resolution were recorded on the MALDI-7090 instrument (Shimadzu Kratos Analytical). A schematic view of the device is presented in Fig.1(B). The instrument is a MALDI-TOF/RTOF instrument but with several noticeable changes from the one previously described: the analyzer dimensions are different, now with a total flight path of 4.1 m, precursor ions are formed using a Nd-YAG laser (λ = 355 nm) pulse rate at 2 kHz, and finally an ASDF cell is located just before the reflectron. Product ions can be obtained with a resolution (full width half maximum, FWHM) of up to 10,000. The instrument is equipped with a curved field reflectron, which allows collection of all product ions, including those generated by metastable/post-source decay (PSD) as well as by CID.

### Materials

The MALDI matrices, α-cyano-4-hydrocinnamic acid (α-CHCA) and 2,5-dihydroxybenzoic acid (DHB), were purchased from Laser Bio Labs (Sophia-Antipolis Cedex, France). Ammonium dihydrogen phosphate (ADHP), acetonitrile (MeCN), trifluoroacetic acid (TFA) and 2,4,6-trihydroxyacetophenone (THAP) were obtained from Sigma-Aldrich (St. Louis, MO, USA). Analytical grade methanol (MeOH) and sodium chloride (NaCl) were purchased from Merck (Darmstadt, Germany). Ethanol (EtOH) was obtained from Rathburn Chemicals (Walkerburn, UK). The ProteoMass P_14_R_1_ MALDI-MS standard and the ether glycerophospholipid 1,2-dihexadecylglycero-phosphatidylcholine, were also acquired from Sigma-Aldrich. Castor bean oil (pharmaceutical grade) containing as its major triglyceride triricinoleoylglycerol was obtained from a local Austrian pharmacy store. Both lipid samples were used without further purification. The complex high mannose oligosaccharide (Man)_8_(GlcNAc)_2_ was supplied by Ludger (Abingdon, UK).

### Sample preparation

The ProteoMass P_14_R MALDI-MS standard was used as the example for peptide analysis. The compound was diluted in 70:30 (v/v) MeCN/H_2_O solution containing 0.1% TFA to give a final concentration of 2 pmol/μL and 0.5 μL was applied to the MALDI target. 0.5 μL of α-CHCA (5 mg/mL in 70:30 (v/v) MeCN/H_2_O solution with 0.1% TFA containing 10 mM ADHP) solution was added to the peptide sample spot. Finally, the sample spot was dried at room temperature.

The lipid sample preparation was performed according to previously published procedures^23^,^24^ by mixing 0.1–1 mg lipid sample/1 mL MeOH with the MALDI-MS matrix solution (1:1, v/v). The matrix solution contained 15 mg THAP, dissolved in 1 mL of MeOH saturated with NaCl. This mixture was sonicated prior to use. For final sample deposition, 0.5 μL of the analyte/matrix mixture was deposited onto the aforementioned MALDI target and dried at room temperature.

The complex high mannose oligosaccharide (Man)_8_(GlcNAc)_2_ was diluted in deionized water, to a concentration of 5 pmol/μL. A solution of 12.5 mg/mL DHB in 50:50 (v/v) MeCN/H_2_O, containing 2.5 mM of NaCl, was used as the matrix solution. For final sample preparation, 0.5 μL of the (Man)_8_(GlcNAc)_2_ solution was deposited onto the described MALDI target and mixed with 0.5 μL of the MALDI matrix solution before being left at room temperature to air dry. Once dry, the sample was recrystallized by means of the addition of 0.2 μL of EtOH and dried again at ambient temperature.

## Results and Discussion

### MS/MS of P14R (peptide)

**Figure 2:**
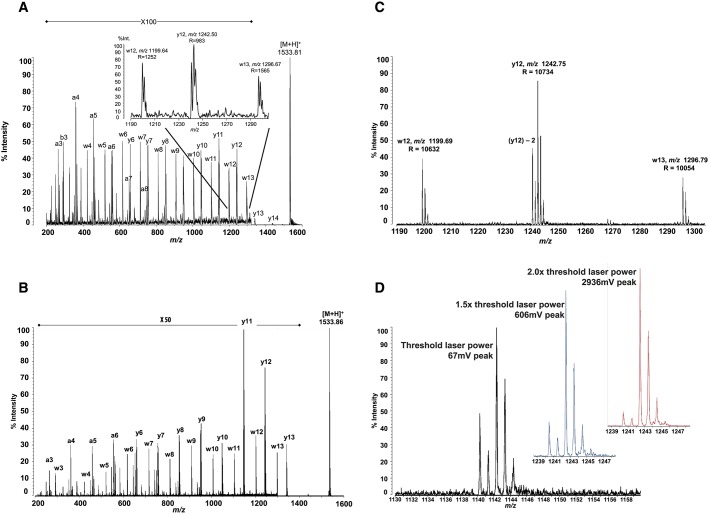
Positive ion mode, high-energy CID of P_14_R_1_ (precursor ion [M + H]^+^ at *m*/*z* 1533.86) without (A) and with (B–D) ASDF: (A) Full range CID spectrum acquired without ASDF. Inset shows expanded *m*/*z* region 1190–1305 (Note: this spectrum was acquired on an Axima Performance MALDI-TOF/RTOF instrument as it is not possible to acquire MS/MS spectra on the MALDI-7090 without ASDF); (B) full range CID spectrum (MALDI-7090 instrument) acquired with ASDF; (C) *m*/*z* region exhibiting w-type product ions (*m*/*z* 1199.69, R = 10632 (FWHM) and *m*/*z* 1296.27, R = 10054 (FWHM)), and a y-type ion (*m*/*z* 1242.75, R = 10734 (FWHM)); (D) *m*/*z* region exhibiting a y_12_- and [y_12_ – 2]-type ion (*m*/*z* 1242.69 and *m*/*z* 1240.69, respectively) at threshold, 1.5 times threshold and 2 times threshold laser power.

The pentadecapeptide, P_14_R_1_ was used to demonstrate the type of product ion spectra produced via high-energy (HE, 20 keV) CID both without ASDF and with ASDF. The latter spectrum (Fig.2(B)) shows the effect of ASDF on the improved resolution of the product ions as well as the precursor ions. The side-chain cleavages (w- and [y–2]-type ions) observed in CID spectra from both instruments are indicative of HE-CID.^25^

Figure2 depicts comparative CID spectra (precursor ion at *m*/*z* 1533.86) taken using the MALDI-7090 (with ASDF) and the Axima Performance instrument (without ASDF). Figure2(A) exhibits a CID spectrum recorded on the Axima Performance (not equipped with an ASDF cell). A y type ion series is observed along with the w-type ion series but with such low resolution that the isotope envelope is hardly visible. Figure2(B) represents the CID spectrum recorded with an instrument equipped with the ASDF device (MALDI-7090), where product ions are detected with higher resolution (approximately 10,000 (FWHM) – for details, see Fig.2(C)). With ASDF, the resolution is such that individual isotopes are resolved to the baseline because peak-widths are around 0.1 *m*/*z* units. The effect of ASDF is a considerable increase in the resolution of all the product ions (y-, w- and a type ion series) but an improved signal-to-noise (S/N) ratio is also achieved (see Table1 for details regarding the increase in resolution). The higher S/N ratio means in the end also higher sensitivity. The magnified region in Fig.2(C) confirms that it was possible to resolve the isotopic envelopes of multiple product ions, with peak resolution obtained in the region of 10,000 FWHM. The product ions w_12_ and w_13_ can be observed with relatively high intensity compared to the y_12_ ion. Ions resulting from side-chain cleavages are typically present due to higher energy collision processes, rather than laser-induced dissociation alone (i.e. when no gas is used). This phenomenon has been described elsewhere^26^,^27^ and a comparison of laser-induced dissociation and HE-CID has also been discussed in an earlier publication.^28^

**Table 1:** Comparison of the resolution for selected product ions of P_14_R peptide and the typical product ion resolution (P_14_R peptide and lipids) with and without ASDF

Product ion or class of compound	Resolution of the product ions without ASDF (AXIMA Performance)	Resolution of the product ions with ASDF (MALDI-7090)
*m*/*z* 1296.7 (w_13_)	1565	10054
*m*/*z* 1242.7 (y_12_)	983	10734
*m*/*z* 1199.7 (w_12_)	1252	10632
Peptide (P_14_R)	Typically 300–1600^a^	Typically 4000–9900^a^
Lipids	Typically 500–1400^a^	Typically 2900–9900^a^

Depending on *m*/*z* range.

The [y_12_–2] product ion, which is observed with intensity similar to that of nearby w ions (w_12_ and w_13_), is also due to high-energy collision processes. The [y_12_–2] ion is particularly interesting in terms of high-energy collision processes and high-resolution CID spectra as such ions could not be seen (or at least resolved properly) in the spectra without ASDF (Fig.2(A)). The description of such ions dates back to classical tandem mass spectra obtained from four-sector instruments.^29^

Further experiments were performed to assess the robustness of this resolution enhancement across varying levels of deposited laser energy. The examples in Fig.2(D) were acquired with increasing laser energy starting at the threshold for desorption/ionization, then 1.5 and 2 times above the threshold. It is reasonable to expect that since the laser energy and, as such, signal intensity increases, peak resolution would decrease.^18^,^30^ However, it can be observed that even when the laser fluence is set to twice that of the threshold for desorption/ionization, monoisotopic resolution is still achieved. This generates improved S/N ratio as well as increased absolute intensity, whilst maintaining resolved isotopic pattern of product ions. An interesting observation could be made when looking at the intensity of the [y_12_-2]-type ion. As the laser energy deposited is increased, the intensity of this ion (in relation to the y_12_-type ion) decreases. This can be explained through the assumption that as we increase the laser energy deposited, the metastable decay pathway (y_12_) becomes “more pronounced” than the high-energy collision pathway [y_12_–2].

### MS/MS of lipids

**Figure 3:**
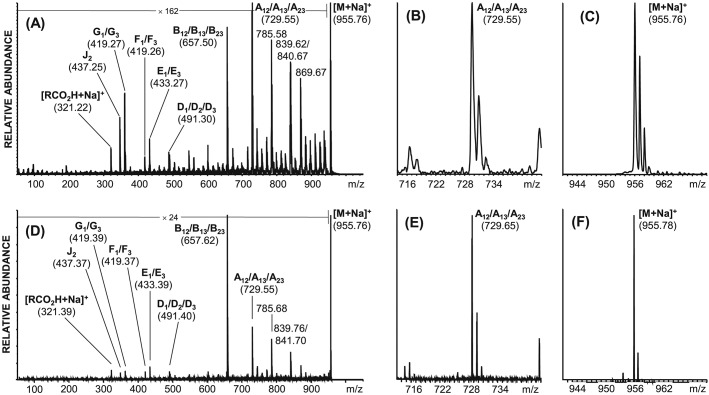
Positive ion mode, high-energy CID of the [M + Na]^+^ ion of triricinoleoylglycerol (*m*/*z* 955.76) using the Axima Performance instrument (A–C) and the MALDI-7090 instrument (D–F): (A) full range CID spectrum; (B) *m*/*z* region exhibiting the A-type product ion (*m*/*z* 729.55, R = 1200 (FWHM)); (C) *m*/*z* region exhibiting the [M + Na]^+^ precursor ion (*m*/*z* 955.76, R = 2200 (FWHM)); (D) full range CID spectrum; (E) *m*/*z* region exhibiting the A-type product ion (*m*/*z* 729.65, R = 9400 (FWHM)); (F) *m*/*z* region exhibiting the [M + Na]^+^ precursor ion (*m*/*z* 955.76, R = 8700 (FWHM)).

The major component of castor bean oil, triricinoleoylglycerol (ricinoleic acid = 12-hydroxyoleic acid), constitutes an ideal compound for testing the fragmentation properties of high-energy (20 keV) CID MALDI TOF/RTOF-MS with helium as collision gas. This is due to the high abundance of charge-remote fragmentation that occurs in high-mass regions. From this mixture triglycerides were desorbed and ionized easily, with the major component generating an abundant sodiated species at *m*/*z* 955.76. Almost no *in-source* fragmentation could be detected under appropriate ion source conditions, i.e. when the deposited laser energy is slightly above the threshold for analyte desorption/ionization. By selecting the aforementioned, dominant precursor ion using a double Bradbury-Nielsen ion gate width of ±3 *m*/*z* units on the Axima Performance instrument,^19^ almost the entire isotope envelope is selected. This yields one product ion, the B-type ion at *m*/*z* 657.50, under typical PSD conditions (data not shown). After introducing helium into the differentially pumped collision cell at a cell pressure of roughly 5 × 10^−6^ mbar the precursor ion is attenuated by approximately 70 to 80% yielding abundant and numerous product ions (see Fig.3) as previously reported.^23^,^31^

In particular, high-mass charge-remote fragmentation of the fatty acid substituents is detected, thus elucidating the position of the hydroxyl group and of the double bond. This is seen in Fig.3(A) where a mass difference of 30 *m*/*z* units (*m*/*z* 869.67 to 839.62) indicates the position of the hydroxy group and a mass difference of 54 *m*/*z* units (*m*/*z* 839.62 to 785.58) indicates the location of the double bond in the fatty acid alkyl chain. Additional product ions include the loss of RCOOH (B-type ion, *m*/*z* 657.50) and the structurally diagnostic E_1/3_- (*m*/*z* 433.27), F_1/3_- (*m*/*z* 419.26), G_1/3_- (*m*/*z* 437.25) and J_2_-type ions (*m*/*z* 437.25). The latter two structurally diagnostic product ions are typically only observed under very high-energy (20 keV) CID conditions.

When using the Axima Performance, the precursor ion resolution is between 2200 and 2900 (FWHM), with product ions typically at 500–1400 resolution depending on the *m*/*z* range (see Figs.3(B) and 3(C)). When selecting the same sodiated triacylglycerol precursor ion as measured with the Axima Performance and fragmenting on the MALDI-7090 (with ASDF applied), a significant improvement in resolution of the precursor ion is observed (see Fig.3(F)) R = 9000–12500 (FWHM)). In addition to this, a dramatic increase in resolution of product ions (R = 2900–9900 (FWHM)) depending on the *m*/*z* range of the product ions is obtained. The overall fragmentation patterns for both tandem TOF instruments appear to be very similar with only minor relative intensity variations. A summary of those results with and without ASDF can be found in Table1.

Another example demonstrating the capabilities of 20 keV collisions with the ASDF implementation for the structural elucidation of lipids is the analysis of the ether glycerophospholipid 1,2-dihexadecylglycerophosphatidylcholine. This lipid easily desorbs/ionizes as a protonated molecule with high abundance and without significant *in-source* fragmentation.

**Figure 4:**
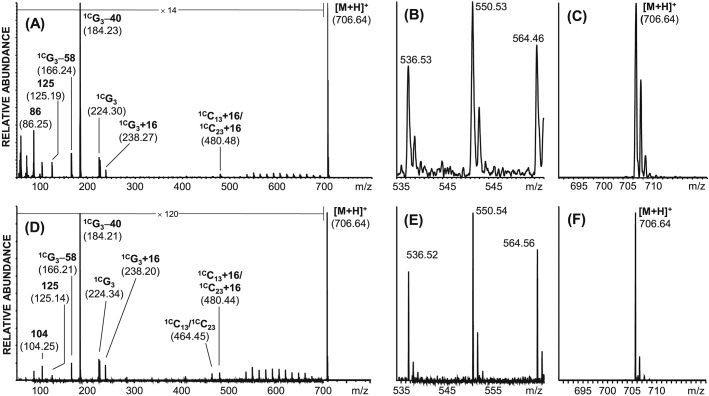
Positive ion mode, high-energy CID MALDI TOF/RTOF-MS of the [M + H]^+^ ion of ether 1,2-dihexadecylglycerophosphatidylcholine (*m*/*z* 706.64) using the Axima Performance instrument (A–C) and the MALDI-7090 instrument (D–F): (A) full range CID spectrum; (B) *m*/*z* region exhibiting charge-remote product ions (*m*/*z* 550.53, R = 1000 (FWHM)); (C) *m*/*z* region exhibiting the [M + H]^+^ precursor ion (*m*/*z* 706.64, R = 1900 (FWHM)); (D) full range CID spectrum; (E) *m*/*z* region exhibiting charge-remote product ions (*m*/*z* 550.54, R = 7500 (FWHM)); (F) *m*/*z* region exhibiting the [M + H]^+^ precursor ion (*m*/*z* 955.76, R = 14200 (FWHM)).

High-energy CID of this precursor ion (*m*/*z* 706.64) shows significant high-mass charge-remote fragmentation with a mass differences of 14 *m*/*z* units for product ions originating from the two hexadecyl alkyl chains (see Figs.4(A) and 4(D)). The highly abundant low-mass ions are characteristic for the phosphatidylcholine polar head group with the diagnostic ions ^1C^G_3_ (*m*/*z* 226), ^1C^G_3_–40 (*m*/*z* 184), ^1C^G_3_-58 (*m*/*z* 166), *m*/*z* 104 (choline) and *m*/*z* 86 (dehydrated choline). The product ion nomenclature used for this analyte group is according to Pittenauer and Allmaier.^24^

Based on these initial comparative measurements on two different types of glycerolipids, the fragmentation patterns obtained with the two instruments are readily comparable. The increase in mass spectrometric resolution for precursor ions is approximately a factor of 4–7.5 (see Figs.3(C), 3(F), 4(C) and 4(F)) and approximately 7.5 for product ions (see Figs.3(B), 3(E), 4(B) and 4(E)). When using the ASDF device (in the MALDI-7090) the identification of neighbouring product ions separated by 1 *m*/*z* unit becomes much easier and as such allows for the straightforward structure-related interpretation of lipid CID spectra in lipidomic projects.

### MS/MS of oligosaccharide

**Figure 5:**
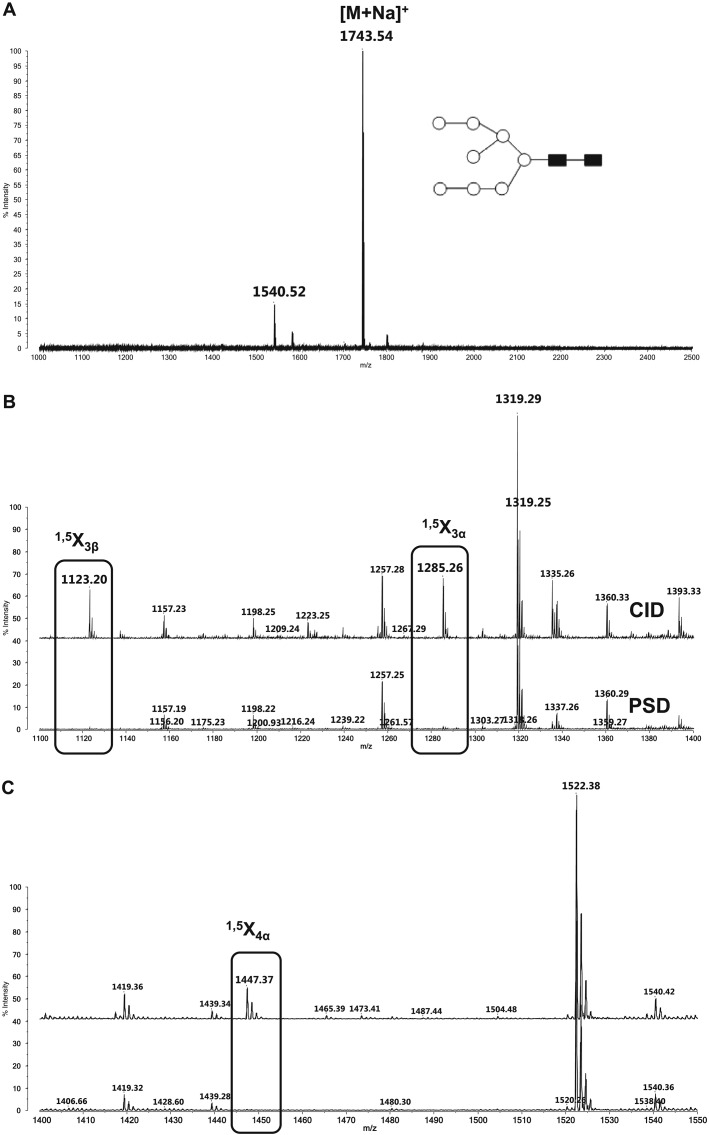
(A) Positive ion mode MALDI mass spectrum of (Man)_8_(GlcNac)_2_ detected as a sodiated adduct ion at *m*/*z* 1743.54. (B) *m*/*z* region (1100–1400) of the MALDI-PSD-MS/MS (bottom) vs MALDI-CID-MS/MS (top) mass spectra where cross-ring cleavages (X_3_) are clearly visible. (C) *m*/*z* region (1400–1500) with PSD-MS/MS (bottom) and CID-MS/MS (top) spectra exhibiting an X_4_ cross-ring cleavage.

The spectrum shown in Fig.5(A) is a MALDI mass spectrum of the Man_8_(GlcNac)_2_ oligosaccharide recorded using the DHB matrix in positive ion mode and detected as a sodiated species [M + Na]^+^. Figures5(B) and 5(C) represent selected *m*/*z* ranges of the MALDI-CID and -PSD spectra of the Man_8_(GlcNac)_2_ sodiated species. Oligosaccharides are usually observed using DHB matrix and are frequently detected as sodiated species.^10^ The CID spectrum of this compound has also been recorded on a MALDI-TOF/RTOF instrument but without an ASDF cell.^19^ In this latter study, it was found that the CID spectra of Man_8_(GlcNac)_2_Na exhibit B- and Y-type ions^32^ mainly when the low-energy collision regime conditions are met. Those types of product ions provide information related to the interglycosidic linkage of the carbohydrate chain. However, in high-energy collision processes, product ions related to cross-ring cleavages lead to more detailed structural information concerning the carbohydrate branching and modifications.^10^ These X-type ions are usually obtained with lower resolution in MALDI-derived CID spectra. This is because the user generally needs to increase the deposited laser energy in order to obtain these product ions. This is especially true with the DHB matrix, which is known to be a so-called ‘cold’ matrix.^33^

In Fig.5(B), it is clearly visible that the product ions ^1,5^X_3β_ and ^1,5^X_3α_ from the CID spectra of the sodiated Man_8_(GlcNac)_2_ molecule, recorded with the MALDI-7090 and ASFD on, are obtained with monoisotopic resolution (spectra on the top). An interesting observation is the relatively lower intensity of this species when recording the PSD spectra using exactly the same conditions (laser fluence, matrix, etc.). The only difference in the CID spectra from the PSD spectrum is the use of helium gas in the differentially pumped collision cell. The same observation is made when looking at another *m*/*z* range (Fig.5(C)). In this latter case the ^1,5^X_4α_ product ion appeared to be much more pronounced in the CID spectrum and is almost not visible in the PSD spectrum. It is important to note that our observation is also valid when we compare the relative abundance of the product ions with each other.

## Conclusions

This paper describes the generation of MALDI-TOF/RTOF spectra with high resolution at “true” high energy using a curved field reflectron in combination with ASDF. CID spectra from a wide range of biomolecules are shown. The CID spectra of two lipids, triricinoleoylglycerol and 1,2-dihexadecaglycerophosphatidylcholine, served to demonstrate that it was possible to generate high-energy and high-resolution tandem TOF spectra. In turn, this allowed the detection of C–C backbone fragmentation processes as well as the location of the hydroxyl group and double bond within the fatty acid moiety with high confidence. The example of the P_14_R_1_ peptide demonstrates the dramatic effect of ASDF in terms of resolution enhancement (in excess of 10,000 (FWHM)) but also the improved signal-to-noise ratio, i.e. tandem spectra containing isotopically resolved product ions with improved sensitivity. The carbohydrate (Man)_8_(GlcNac)_2_ was chosen for the comparison of CID and PSD spectra when using ASDF. The CID spectra exhibited not only linkage-related product ions (Y- and B-type ions), but also cross-ring cleavage product ions (X-type ions).
